# Ineffective Ventilation in A Neonate with A Large Pre-Carinal Tracheoesophageal Fistula and Bilateral Pneumonitis-Microcuff Endotracheal Tube to Our Rescue!

**DOI:** 10.21699/jns.v6i1.410

**Published:** 2017-01-01

**Authors:** Anju Gupta, Nishkarsh Gupta

**Affiliations:** 1 Department of Anesthesiology, CNBC, Delhi, India; 2 Department of Anesthesiology, DRBRAIRCH, All India Institute of Medical Sciences, New Delhi, India

**Keywords:** Microcuff tube, Tracheo-esophageal fistula, Esophageal atresia, Airway management

## Abstract

Tracheoesophageal fistula (TEF) is one of the most common congenital anomaly requiring surgical correction in neonatal period. The important goal of airway management is to avoid excessive gastric distension and ensure adequate ventilation prior to surgical ligation of the fistula. If a large fistula is present close to carina, excessive loss of delivered tidal volume may lead to ineffective ventilation. In addition, gastric distension elevates diaphragm and diminishes the lung compliance. If lung compliance is already impaired due to pre-existing lung pathology, situation becomes much more demanding. We report the successful airway management of a patient with large precarinal fistula and bilateral pneumonitis using the novel Microcuff tube. The unique design of microcuff makes it suitable to be used for this purpose. To the best of our knowledge, the use of microcuff ETT for perioperative airway management in case of a large precarinal fistula in a neonate with respiratory pathology has not been reported in the past.

## CASE REPORT

A, 8-day old full term male baby (birth weight 3.1kg) with history of choking, coughing, cyanosis, presented for surgical repair of TEF with EA. He was lethargic, tachypnoeic, dehydrated and had respiratory distress with bilateral coarse crepts. He was kept nil per orally in 45 degree head up position, received broad spectrum antibiotics, asthalin nebulisation, oxygen by hood, intermittent CPAP and intravenous fluids. Thereafter, he was posted for emergency surgery on day 11 of life. He still had bilateral coarse crepts, decreased air entry in left lower zone, a SPO2 of 87-88% on room air which improved to 93-94% on oxygen. Preoperative investigations revealed leuckocytosis, thrombocytopenia (90,000/µl³), elevated blood urea and serum bilirubin. CXR showed wedge shaped opacities involving left lower lobe and right mid zone. After preoxygenation for 3minutes, anaesthesia was induced with inj thiopental sodium 10mg and fentanyl 5µg. The trachea was intubated with 3.5 mm ID uncuffed PVC endotracheal tube (ETT) after achieving neuromuscular blockade with rocuronium 3mg. The ETT was intentionally advanced into right mainstem bronchus and then withdrawn till breath sounds were heard in left lung. But a large air leak was evident into the stomach and the lung compliance and SPO2 (92% with 100% oxygen) worsened. This persisted despite rotating the bevel of ETT anteriorly and repeating the procedure. At this point, a 3.0mm ID microcuff ETT (METT) (Kimberly-Clark, unomedical sdn, Kedah, Malaysia) was inserted in the same manner and placed so that air entry was present in the left lung. The cuff of the ETT was inflated in small increments to total volume of 1ml. This enabled the ventilation of lungs at desired airway pressures without excessive stomach distension for the rest of the surgery. Precordial stethoscope was secured to left axilla to detect inadvertent right endobronchial migration of the tube. METT Cuff pressures were monitored regularly and remained below 15 mm Hg. A mixture of O2 in air was used to achieve a SpO2 more than 90%. On thoracotomy and dissection of fistula, surgeon discovered a large fistula (approx. 6-7mm diameter) within 1cm of the carina. At the end of the case, the METT cuff was deflated and withdrawn to midtracheal position before shifting the child to ICU. Two days later, the baby was extubated and put on nasal CPAP.

## DISCUSSION

Present case demonstrates the successful occlusion of a large precarinal fistula for TEF surgery by use of Microcuff ETT for the first time. In majority of TEF with EA the distal esophagus communicates with posterior wall of trachea and upper part ends in a blind pouch (Type-C).[1] In 22% of such cases the distal fistula lies within 1cm of the carina and can distend stomach if not bypassed to prevent air leak.[1] Conventionally, following endobronchial intubation, the tip of the ETT is withdrawn into the trachea till breath sounds are heard on the left side to ensure minimum air leak. This strategy suffices in most of the cases as the posterior wall of the ETT occludes fistula. Since our patient had a large fistula near carina (defined as >3mm)[2] along with poor lung compliance (due to pneumonitis), use of a conventional uncufffed ETT failed to prevent excessive air leak into the stomach and consequent desaturation. METT has an ultrathin polyurethane cuff (10 μm) which is distally placed along the shaft and provides effective tracheal seal at low pressures.[3] Its cuff expands evenly and does not form folds and channels between the cuff and the tracheal wall. It has been safely used in infants and small children.[4] In a multi-centre study by Weiss et al, trachea was completely sealed in 95% of children (0-5years) using METT the with a cuff pressure of less than 15 cm H2O (mean cuff pressure 9.7 cm H2O).[4] In our case also, the cuff pressures were maintained below 15 cm H2O during surgery. It has also been used in preterm infant with TEF for achieving effective mechanical ventilation in ICU for 5 days without any obvious tracheal morbidity.[5] Occlusion of fistula for TEF repair with conventional PVC cuffed ETT has been reported previously.[6] A METT doesn’t have a Murphy’s eye and has a distally located cuff (5mm from the tip in 3.0mm ID ETT, Fig 1), so we considered it a better option to occlude a precarinal fistula. Occlusion of fistula with fogarty catheter/ endobronchial blocker under Rigid/ fiberoptic tracheoscopy has also been described previously.[2,7] Rigid bronochoscopy is not routinely done at our setup prior to TEF repair and our only neonatal fiberoptic bronchoscope was non-functional, hence this option was not considered. Maintenance of spontaneous respiration till the fistula is ligated has been suggested to avoid gastric distension during positive pressure ventilation.[2] But in our patient, due to pre-existing lung pathology it seemed impossible to achieve adequate ventilation without muscle relaxation during thoracotomy in lateral position. Another possible alternative is left endobronchial intubation which has been suggested to bypass the fistula and improve surgical conditions for right sided thoracotomy.[8] However, need for fiberoptic bronochoscopy for left endobronchial intubation and potential risk of desaturation with one lung ventilation deterred us from choosing this option.

In conclusion, METT can be safely and effectively used to occlude large distal TEF because of its suitable design for the purpose. We advocate the need for further large scale research to conclusively prove the same.

**Figure:**
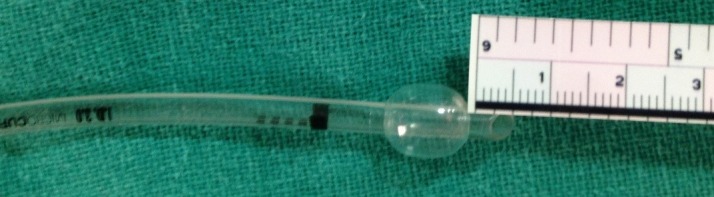
Figure 1: 3.0mm ID METT with Cuff position 5mm from the distal end

## Footnotes

**Source of Support:** Nil

**Conflict of Interest:** Nil

## References

[1] Holzki J. Bronchoscopic findings and treatment in congenital tracheo-oesophageal fistula. Paediatr Anaesth. 1992; 2:297-303.

[2] Andropoulos DB, Rowe RW, Bets JM. Anaesthetic and surgical airway management during trachea-oesophageal fistula repair. Paediatr Anaesth. 1998; 8:313-19. 10.1046/j.1460-9592.1998.00734.x 9672929

[3] Dullenkopf A, Gerber AC, Weiss M. Fit and seal characteristics of a new paediatric tracheal tube with high-volume-low pressure polyurethane cuff. Acta Anaesthesiol Scand. 2005; 49:232–7. 10.1111/j.1399-6576.2005.00599.x 15715626

[4] Weiss M, Dullenkopf A, Fischer JE, Keller C, Gerber AC. Prospective randomized controlled multi-centre trial of cuffed or uncuffed endotracheal tubes in small children. Br J Anaesth. 2009; 103:867–73. 10.1093/bja/aep290 19887533

[5] Famira KM, Schulzke S, Hammer J. Cuffed endotracheal tube for occlusion of a trachea-oesophageal fistula in an extremely low birth weight infant. Intensive Care Med. 2004; 30:1249. 10.1007/s00134-004-2249-x 15085321

[6] Greemberg L, Fisher A, Katz A. Novel use of neonatal cuffed tracheal tube to occlude tracheo-oesophageal fistula. Pediatr Anaesth. 2009; 9:339–41. 10.1046/j.1460-9592.1999.00360.x 10411771

[7] Hammer GB. Pediatric thoracic anesthesia. Anesthesiol Clin North Am. 2002; 20:153-80. 10.1016/s0889-8537(03)00059-2 11892503

[8] Tercan E, Sungun MB, Boyaci A, Kucukaydin M. One lung ventilation of a preterm newborn during esophageal atresia and tracheoesophageal fistula repair. Acta Anaesthesiol Scand. 2002; 46:332-3. 10.1034/j.1399-6576.2002.t01-1-460318.x 11939927

